# Prohibitin 1 Modulates Mitochondrial Stress-Related Autophagy in Human Colonic Epithelial Cells

**DOI:** 10.1371/journal.pone.0031231

**Published:** 2012-02-17

**Authors:** Arwa S. Kathiria, Lindsay D. Butcher, Linda A. Feagins, Rhonda F. Souza, C. Richard Boland, Arianne L. Theiss

**Affiliations:** 1 Division of Gastroenterology, Department of Internal Medicine, Baylor Research Institute, Baylor University Medical Center, Dallas, Texas, United States of America; 2 Institute of Biomedical Studies, Baylor University, Waco, Texas, United States of America; 3 Department of Medicine, Veterans Affairs North Texas Health Care System, University of Texas Southwestern Medical Center, Dallas, Texas, United States of America; Catholic University Medical School, Italy

## Abstract

**Introduction:**

Autophagy is an adaptive response to extracellular and intracellular stress by which cytoplasmic components and organelles, including damaged mitochondria, are degraded to promote cell survival and restore cell homeostasis. Certain genes involved in autophagy confer susceptibility to Crohn's disease. Reactive oxygen species and pro-inflammatory cytokines such as tumor necrosis factor α (TNFα), both of which are increased during active inflammatory bowel disease, promote cellular injury and autophagy via mitochondrial damage. Prohibitin (PHB), which plays a role in maintaining normal mitochondrial respiratory function, is decreased during active inflammatory bowel disease. Restoration of colonic epithelial PHB expression protects mice from experimental colitis and combats oxidative stress. In this study, we investigated the potential role of PHB in modulating mitochondrial stress-related autophagy in intestinal epithelial cells.

**Methods:**

We measured autophagy activation in response to knockdown of PHB expression by RNA interference in Caco2-BBE and HCT116 WT and p53 null cells. The effect of exogenous PHB expression on TNFα- and IFNγ-induced autophagy was assessed. Autophagy was inhibited using Bafilomycin A_1_ or siATG16L1 during PHB knockdown and the affect on intracellular oxidative stress, mitochondrial membrane potential, and cell viability were determined. The requirement of intracellular ROS in siPHB-induced autophagy was assessed using the ROS scavenger *N*-acetyl-*L*-cysteine.

**Results:**

TNFα and IFNγ-induced autophagy inversely correlated with PHB protein expression. Exogenous PHB expression reduced basal autophagy and TNFα-induced autophagy. Gene silencing of PHB in epithelial cells induces mitochondrial autophagy via increased intracellular ROS. Inhibition of autophagy during PHB knockdown exacerbates mitochondrial depolarization and reduces cell viability.

**Conclusions:**

Decreased PHB levels coupled with dysfunctional autophagy renders intestinal epithelial cells susceptible to mitochondrial damage and cytotoxicity. Repletion of PHB may represent a therapeutic approach to combat oxidant and cytokine-induced mitochondrial damage in diseases such as inflammatory bowel disease.

## Introduction

Autophagy is an evolutionarily conserved catabolic pathway that degrades cytoplasmic components such as long-lived proteins, macromolecules and damaged organelles including the endoplasmic reticulum and mitochondria through lysosomal degradation [1], [2], [3]. Autophagy is an adaptive response to extracellular stress, such as starvation, or intracellular stress, including the accumulation of misfolded proteins, damaged organelles, or the invasion of microorganisms, intended to promote cell survival and restore cell homeostasis [4]. Either apoptosis or autophagic cell death can be initiated in irreversibly damaged cells [5]. Malfunctioning autophagy has been associated with multiple diseases such as cancer, neurodegeneration, autoimmune diseases and inflammatory diseases, including inflammatory bowel disease (IBD) [6].

The two common, but disparate, forms of IBD, Crohn's disease and ulcerative colitis, share related characteristics such as mucosal damage and diarrhea but have distinguishing clinical features. The etiopathogenesis of IBD remains unknown but is thought to involve a combination of genetic and non-genetic risk factors that regulate mucosal immune response, mucosal barrier function, and response to microbial factors [7]. Multiple epithelial molecules have been identified as mediators of IBD pathogenesis including those that control epithelial homeostasis [8]. Genome-wide association studies and meta-analysis have identified the autophagy genes ATG16L1, IRGM, and LRRK2 as candidate loci involved in genetic susceptibility to Crohn's disease [9], [10], [11], [12]. Mutation or deletion of ATG16L1 results in increased pro-inflammatory cytokine production, increased susceptibility to experimental colitis, and reduced capability to eradicate invading bacteria, indicating the importance of autophagy in suppressing intestinal inflammation [13], [14], [15].

Multiple studies have reported mitochondrial dysfunction in Crohn's disease and ulcerative colitis [16], [17], [18], [19] as well as the dextran sodium sulfate and 2,4,6-trinitrobenzene sulfonic acid models of colitis [20], [21]. Mitochondria are important regulators of autophagy and apoptosis. During normal function of the mitochondrial respiratory chain, reactive oxygen species (ROS), which are partially reduced oxygen species such as superoxide radical (O_2_^−^), hydrogen peroxide (H_2_O_2_), hydroxyl radical (⋅OH), and peroxynitrate (NOO^−^), are generated at low levels. Production of ROS is increased when mitochondria are damaged [22]. IBD is associated with increased ROS and decreased antioxidant enzymes in the intestinal mucosa [23], [24], [25], [26]. It is widely accepted that ROS produced as a by-product of respiration as well as exogenous ROS can induce autophagy via mitochondrial damage [27], [28]. Mitochondria are the main source of ROS for regulation of autophagy [29]. In fact, exogenous ROS and the proinflammatory cytokine tumor necrosis factor α (TNFα), both of which are increased during IBD, promote cellular injury and autophagy via mitochondrial ROS generation [29], [30], [31]. Defects in autophagy result in the accumulation of intracellular ROS and deformed mitochondria [14], [32].

Prohibitin 1 (PHB) is an evolutionarily conserved, multifunctional 32 kDa protein implicated in cellular processes including the regulation of proliferation, apoptosis, and transcription [33], [34], [35], [36]. PHB is predominantly localized to the mitochondria in intestinal epithelial cells [37] and multiple studies have shown that PHB plays a role in maintaining normal mitochondrial function and morphology (reviewed in [38]). It has been shown that PHB interacts with complex I and subunits of cytochrome *c* oxidase of the respiratory chain and regulates their assembly [39], [40]. Loss of PHB in mitochondria impairs function of the mitochondrial respiratory chain [39], [40]. One obvious effect of respiratory chain dysfunction is increased oxidant production leading to oxidative stress, which can cause alterations in mitochondrial morphology and membrane potential [29].

Expression of PHB is decreased in mucosal biopsies from ulcerative colitis and Crohn's disease afflicted patients and in animal models of colitis [37], [41]. Pro-inflammatory cytokines such as TNFα and oxidative stress induced by exogenous H_2_O_2_ decrease expression of intestinal epithelial PHB in vivo and in vitro [37], [42]. Restoration of colonic epithelial PHB expression using genetic manipulation (villin-PHB transgenic mice) or therapeutic delivery to the colon via nanoparticle or adenovirus protected mice from experimental colitis [43], [44]. Our recent data suggest that epithelial PHB sustains anti-oxidant expression [44] and has anti-inflammatory properties such as reducing TNFα-stimulated NF-κB activation [42]. This is in agreement with emerging data that suggest a role of PHB in combating oxidative stress in multiple cells types [39], [40], [45], [46]. In this study, we investigated the potential role of PHB in modulating mitochondrial stress-related autophagy in intestinal epithelial cells. Here, we show that TNFα and IFNγ-induced autophagy inversely correlates with PHB protein expression and that gene silencing of PHB induces mitochondrial autophagy via increased intracellular ROS. Inhibition of autophagy during PHB knockdown exacerbates mitochondrial depolarization and reduces cell viability. These data suggest that decreased PHB levels coupled with dysfunctional autophagy renders intestinal epithelial cells susceptible to mitochondrial ROS and cytotoxicity.

## Results

### PHB protein expression inversely correlates with cytokine-induced autophagy in cultured colonic epithelial cells

**Figure 1:**
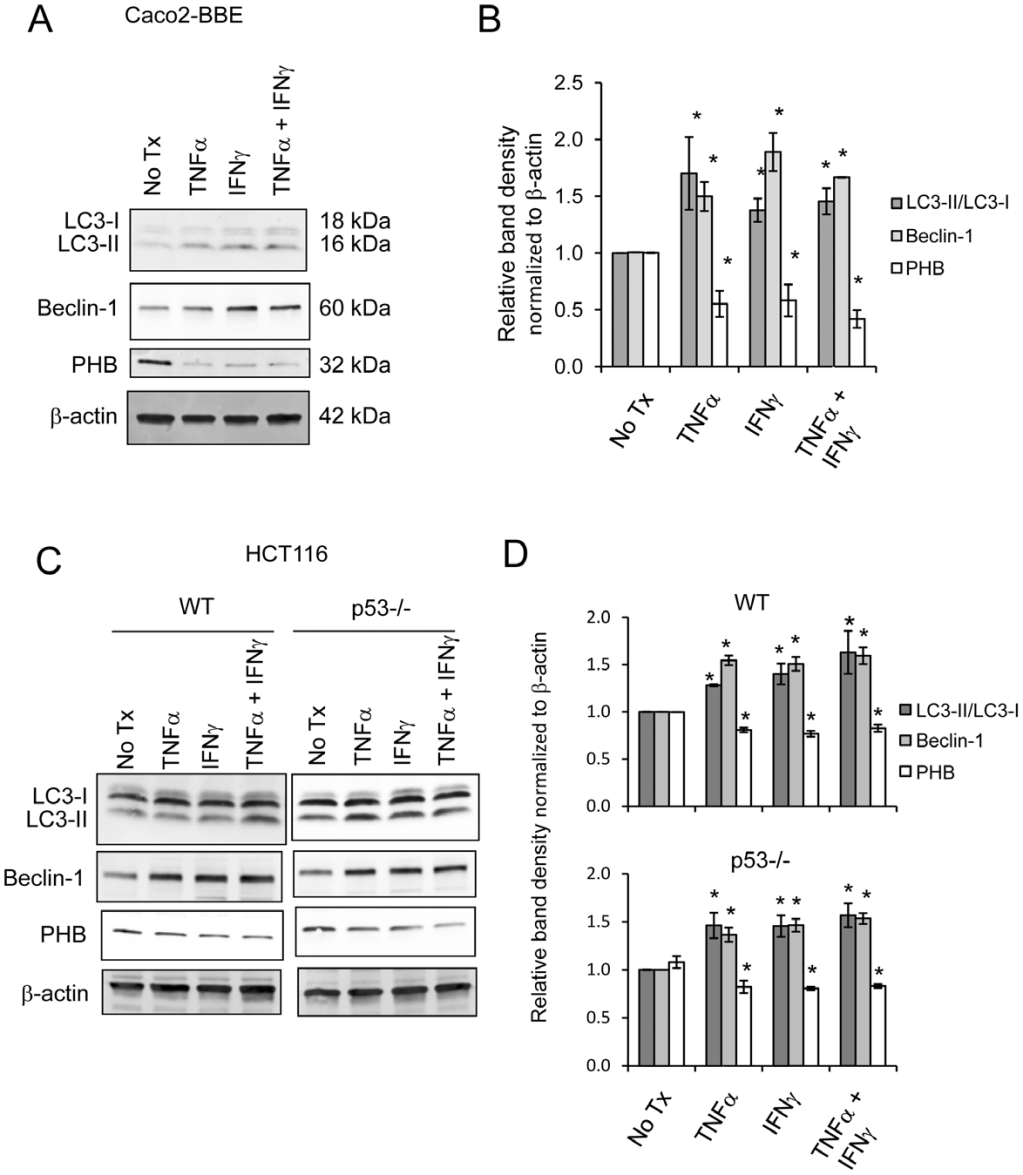
PHB protein levels inversely correlate with cytokine-induced autophagy in cultured colonic epithelial cells. (A) Representative Western blots showing LC3I/LC3II, Beclin-1, PHB or β-actin (loading control) expression in Caco2-BBE cells treated with 10 ng/ml TNFα or 50 ng/ml IFNγ alone or in combination for 18 hours. (B) Histograms show mean ± SEM relative to no treatment control Caco2-BBE cells. *P<0.05 vs. no tx; n = 3. (C) Representative Western blots showing LC3I/LC3II, Beclin-1, PHB or β-actin (loading control) expression in HCT116 cells treated with 10 ng/ml TNFα or 50 ng/ml IFNγ alone or in combination for 18 hours. (D) Histograms show mean ± SEM relative to no treatment control HCT116 cells. *P<0.05 vs. no tx; n = 3.

Our previous studies showed that TNFα reduces expression of PHB in intestinal epithelial cells in vivo and in vitro [42]. Pro-inflammatory cytokines such as TNFα and IFNγ have been shown to induce autophagy in human intestinal epithelial cell lines [31], [47]. Confluent monolayers of Caco2-BBE cells were treated with 10 ng/ml TNFα or 50 ng/ml IFNγ alone or in combination for 18 hours. As expected, TNFα and IFNγ increased two biochemical signs of autophagy: the conversion of LC3-I to LC3-II, indicated by normalizing LC3-II to LC3-I protein levels, and increased beclin-1 protein expression (Figure 1A and 1B) [27], [48]. Conversely, PHB protein levels in the same samples were decreased by TNFα and IFNγ (Figure 1A and 1B). The effect of TNFα and IFNγ given in combination reflected that of cells treated with either cytokine alone and therefore, we did not pursue the effects of these cytokines in combination. It is widely accepted that the tumor suppressor p53 regulates autophagy [49]. Since Caco2-BBE cells have mutated p53 [50], we assessed the involvement of p53 in autophagy induction by TNFα and IFNγ in wild-type (WT) and p53 null HCT116 colonic epithelial cells. HCT116 cells, including p53 null cells [51], also showed the conversion of LC3-I to LC3-II and increased beclin-1 protein expression suggesting that the effect of TNFα and IFNγ to increase autophagy and decrease PHB protein expression is independent of p53 signaling (Figure 1C and 1D).

### Exogenous PHB expression reduces basal autophagy and TNFα-induced autophagy in intestinal epithelial cells

**Figure 2:**
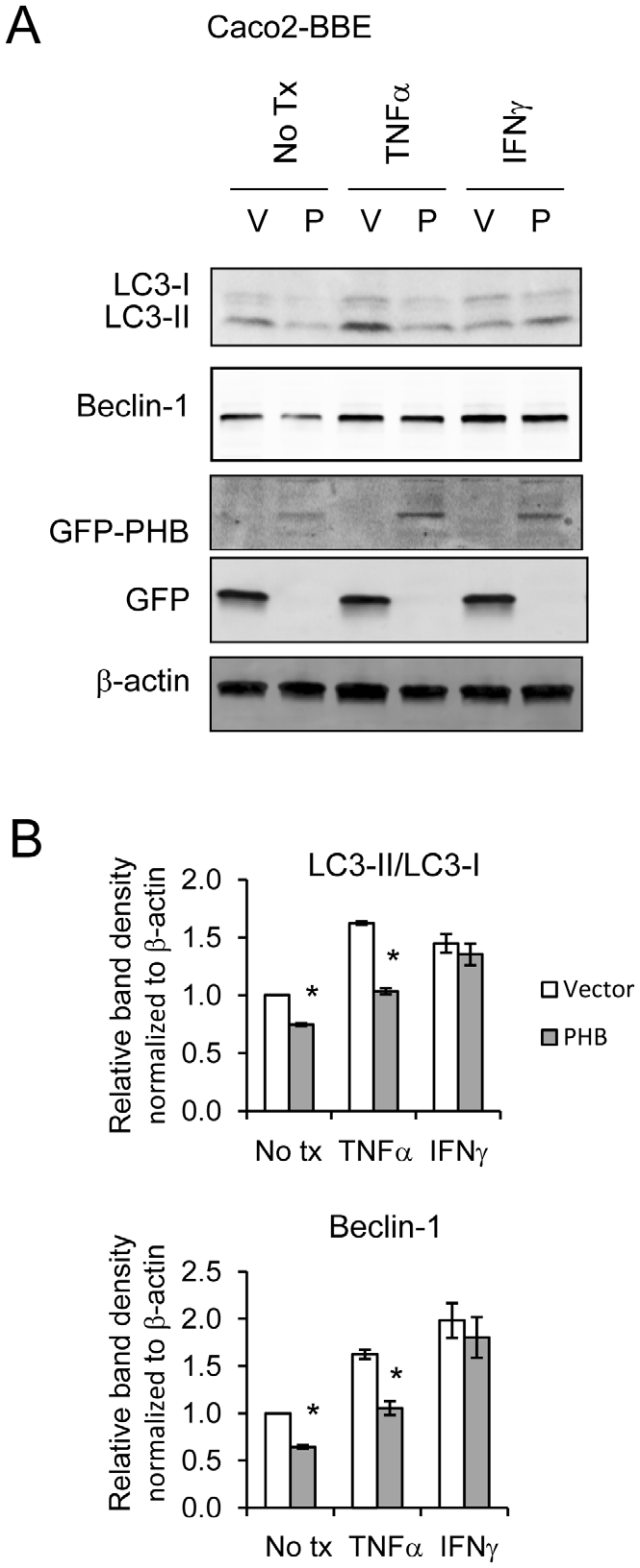
Exogenous PHB expression reduces basal autophagy and TNFα-induced autophagy in intestinal epithelial cells. (A) Representative Western blots of 3 independent experiments showing LC3I/LC3II, Beclin-1, GFP-PHB, GFP or β-actin (loading control) expression in Caco2-BBE cells transfected with either pEGFPN1 expression vector (V) or pEGFPN1-PHB (P) and treated with 10 ng/ml TNFα or 50 ng/ml IFNγ for 18 hours. (B) Histograms show mean ± SEM relative to no treatment control cells. *P<0.05 vs. no tx; n = 3.

Since PHB expression inversely correlated with the induction of autophagy in colonic epithelial cells, we determined whether exogenous PHB expression could affect autophagy. Caco2-BBE cells stably overexpressing GFP-tagged PHB (pEGFPN1-PHB) show decreased LC3-II conversion from LC3-I and reduced beclin-1 protein expression (Figure 2A and 2B), suggesting a reduction in basal autophagy. Treatment with TNFα or IFNγ increased LC3-II and beclin-1 protein abundance in empty vector expressing cells (Figure 2A and 2B), reflecting the same response as WT Caco2-BBEs in Figure 1A. PHB overexpressing cells showed decreased TNFα-induced LC3-I conversion to LC3-II and beclin-1 protein expression compared to vector-transfected cells, whereas PHB overexpression did not affect IFNγ-induced LC3-II or beclin-1 protein expression (Figure 2A and 2B). Since PHB overexpression did not decrease IFNγ-induced autophagy, this would suggest that the IFNγ autophagy pathway is distinct from that of TNFα. An antibody specific to GFP was used to assess protein expression of GFP-PHB and GFP in PHB and vector overexpressing cells, respectively.

### Knockdown of PHB induces autophagy

**Figure 3:**
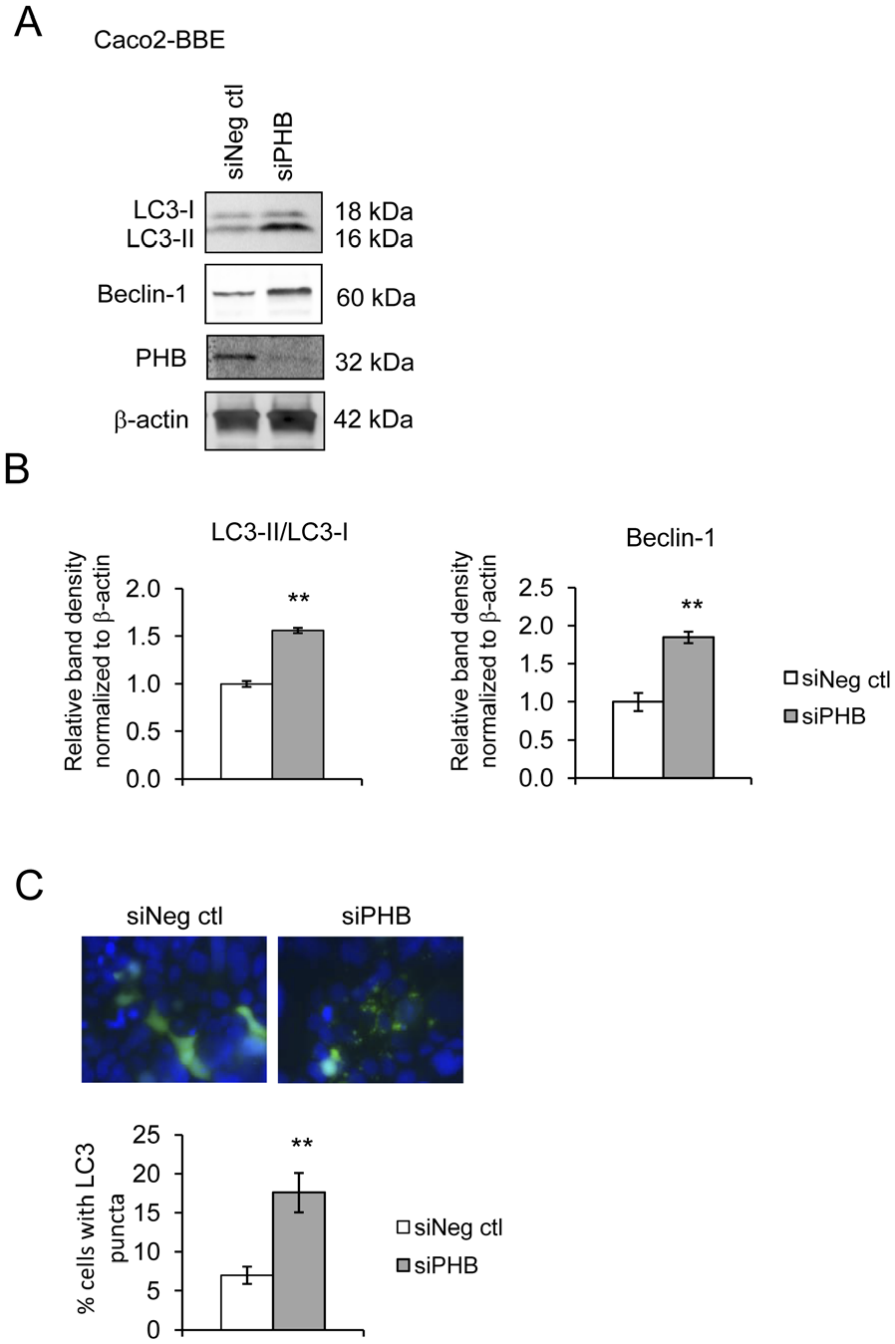
Knockdown of PHB induces autophagy. Markers of autophagy were assessed in Caco2-BBE cells transfected with either siNeg ctl or siPHB for 96 hours. (A) Representative Western blots showing LC3I/LC3II, Beclin-1 or β-actin (loading control) expression. PHB protein levels were assessed to determine efficiency of knockdown. (B) Histograms show mean ± SEM. **P<0.01 vs. siNeg ctl; n = 3 (C) Fluorescent micrographs of cells expressing pSelect-GFP-LC3 (green) and stained with DAPI (blue) to visualize nuclei. Quantification of cells with punctuate GFP-LC3. Histograms show mean ± SEM. **P<0.01 vs. siNeg ctl, n ≥ 5 fields with >50 cells/field.

Since PHB protein expression inversely correlated with TNFα- and IFNγ-induced autophagy in Caco2-BBE cells, we next determined whether knockdown of PHB expression could induce autophagy. Caco2-BBE cells transfected with siPHB showed conversion of LC3-I into LC3-II (Figure 3A) as well as increased beclin-1 protein expression (Figure 3B) compared to cells transfected with a negative control siRNA (siNeg ctl). PHB knockdown stimulated the redristribution of GFP-LC3 fusion protein from a diffuse signal to cytoplasmic puncta indicative of autophagosomes (Figure 3C). The mitochondrial stress proteins Cpn60, PKR, and ClpP showed no change in expression by Western blot upon PHB knockdown suggesting that inhibition of PHB does not induce the mitochondrial unfolded protein response (Figure S1). The efficiency of siRNA knockdown was validated by western blot (Figure 3A). Protein expression of PHB was reduced ∼80% 96 hours after transfection.

**Figure 4:**
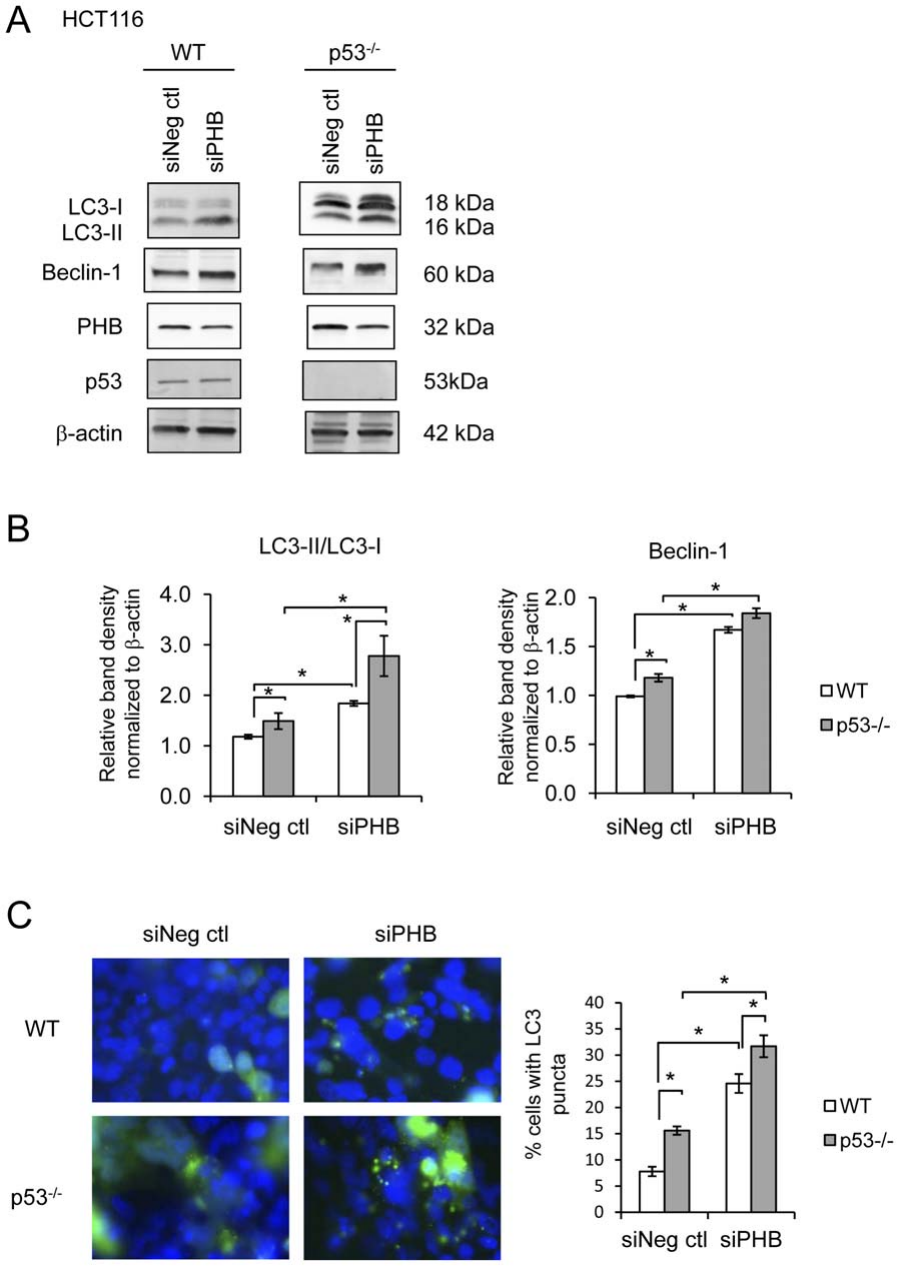
Knockdown of PHB using siRNA induces autophagy independently of p53 in HCT116 cells. Markers of autophagy were assessed in HCT116 cells transfected with either siNeg ctl or siPHB for 96 hours. (A) Representative Western blot showing LC3I/LC3II , Beclin-1 or β-actin (loading control) expression. PHB protein levels were assessed to determine efficiency of knockdown. (B) Histograms show mean ± SEM relative to no treatment control cells. *P<0.05 vs. siNeg ctl; n = 3. (C) Fluorescent micrographs of cells expressing pSelect-GFP-LC3 (green) and stained with DAPI (blue) to visualize nuclei. Quantification of cells with punctuate GFP-LC3. Histograms show mean ± SEM. *P<0.05 vs. siNeg ctl, n = 5 fields with >50 cells/field.

The effect of PHB knockdown in Caco2-BBE cells was corroborated in HCT116 cells. Knockdown of PHB induced the conversion of LC3-I to LC3-II, increased beclin-1 protein expression (Figure 4A and 4B) and increased the formation of cytoplasmic puncta by the GFP-LC3 fusion protein (Figure 4C). Although knockdown of PHB in HCT116 cells (approximately 50% reduction compared to control; Figure 4A) was not as strong as in Caco2-BBE cells, the results on autophagy were similar to that in Caco2-BBE cells. p53 inhibition, including p53 knockout by homologous recombination, initially induces autophagy by ER stress followed by mitophagy (autophagy of mitochrondria) [52]. To determine whether the induction of autophagy by PHB knockdown is dependent on p53, markers of autophagy were assessed in p53^−/−^ HCT116 cells transfected with siPHB. p53^−/−^ cells showed increased LC3-II and GFP-LC3 puncta formation compared to WT cells (Figure 4A and 4C) as previously described [52]. p53^−/−^ cells also exhibited increased beclin-1 protein expression compared to WT cells (Figure 4A and 4B). Knockdown of PHB caused a further increase in autophagy in p53^−/−^ cells, suggesting that siPHB-mediated autophagy is independent of p53 signaling. ER stress is not further increased in p53^−/−^ cells upon PHB knockdown (Figure S2).

### PHB knockdown increases intracellular reactive oxygen species and induces mitochondrial depolarization

**Figure 5:**
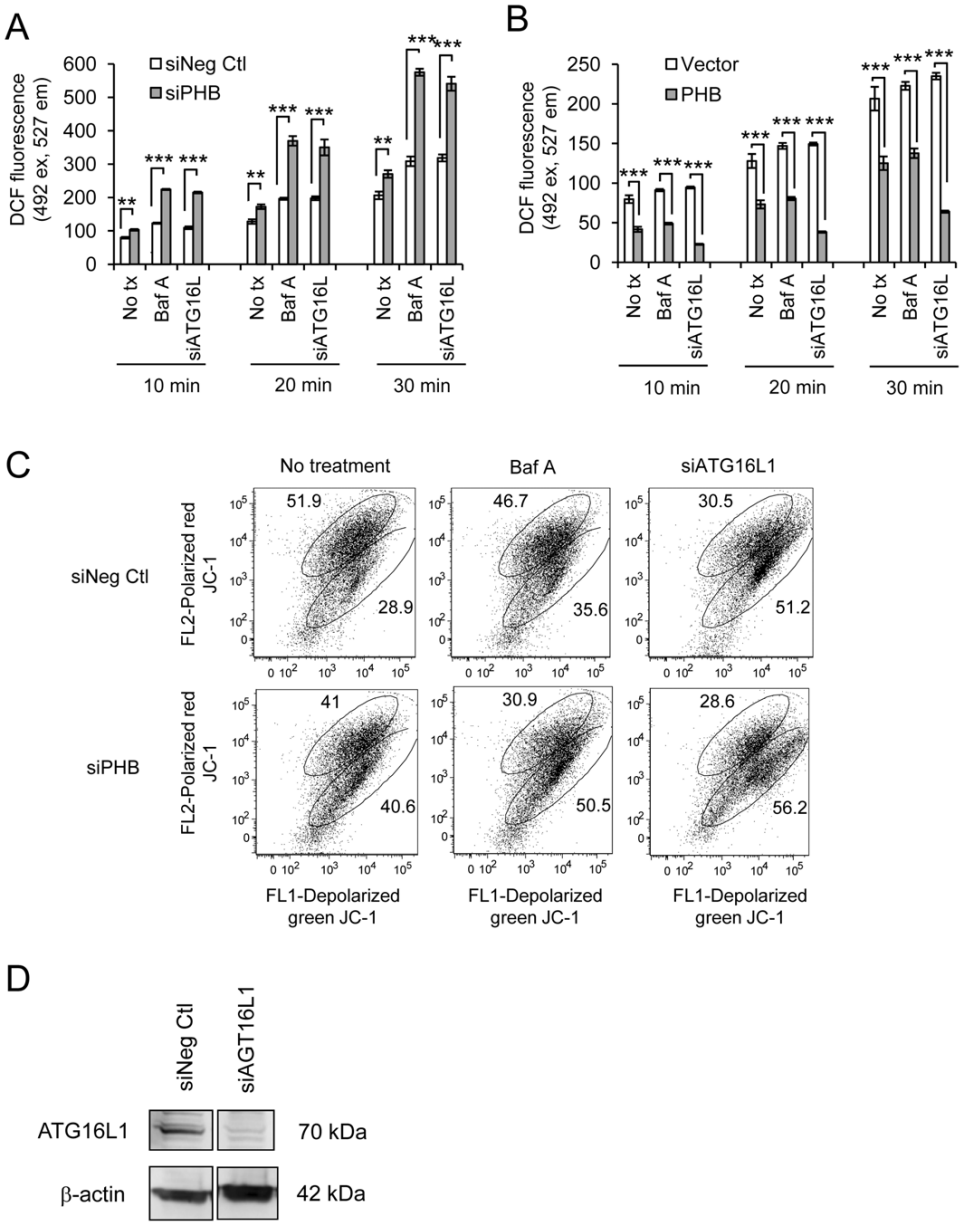
PHB knockdown increases intracellular reactive oxygen species and induces mitochondrial depolarization. (A) DCF fluorescence was measured in Caco2-BBE cells transfected with siRNA for 96 hours and treated as indicated for 18 hours. Cells were loaded with DCFH-DA for 10 minutes and fluorescence was determined 10, 20 and 30 minutes after loading using a plate reader to assess intracellular ROS levels. **P<0.01, ***P<0.001; n = 16 from two separate experiments. (B) DCF fluorescence was measured in Caco2-BBE cells stably transfected with pEGFPN1 expression vector or pEGFPN1-PHB as in (A). ***P<0.001; n = 8. (C) Mitochondrial membrane depolarization was measured in Caco2-BBE cells stained with JC-1 dye as demonstrated by a shift from red (intact MMP) to green (depolarized) fluorescence detected by flow cytometry. The data shown are a representative result from one of three experiments. (D) ATG16L1 protein levels were assessed by Westen blotting to determine efficiency of knockdown.

Autophagy is induced by multiple stimuli including damage to organelles such as the endoplasmic reticulum and mitochondria. Given that the predominant subcellular localization of PHB is in the mitochondria in most cell types studied to date, emerging data suggest that PHB plays a role in stabilizing components of the electron transport chain [38]. Depletion of PHB function has been shown to cause electron transport chain disruption and increased intracellular oxidative stress [39], [40]. As an indicator of intracellular ROS, Caco2-BBE cells transfected with siPHB or siNeg ctl were loaded with the oxidation-sensitive dye H_2_DCF-DA and assayed for DCF fluorescence 10, 20 and 30 minutes after loading using a plate reader. PHB knockdown caused a 30% increase in intracellular ROS compared to control cells at each time point measured (Figure 5A; No tx). Recent data has shown that specific gene mutations or deficiency in autophagy is linked to susceptibility to inflammatory bowel disease [9], [10], [11], [12]. To mimic this situation, autophagy was inhibited using 100 nM Baf A for 12 hours or RNAi against the autophagy gene ATG16L1. Efficiency of ATG16L1 knockdown is shown in Figure 5D. Inhibition of autophagy increased intracellular ROS in both control and siPHB-transfected cells; the magnitude, however, was significantly greater in siPHB-transfected cells at all time points (Figure 5A; Baf A and siATG16L). Caco2-BBE cells stably overexpressing PHB show reduced DCF fluorescence compared to vector-transfected cells (Figure 5B; No tx). In the setting of PHB overexpression coupled with autophagy inhibition (Baf A or siATG16L1), ROS levels are not further increased and in fact are decreased during loss of ATG16L1. These results suggest that PHB expression regulates intracellular ROS and that autophagy prevents the accumulation of ROS.

To determine whether the increase in intracellular ROS is associated with altered mitochondrial integrity, mitochondrial membrane potential (MMP) was assessed using JC-1 dye. As shown in Figure 5C, the percentage of Caco2-BBE cells testing positive for depolarized mitochondria increased from ∼29% of control cells to ∼41% of cells with PHB knockdown in the absence of inhibitors of autophagy (No tx, left panels), suggesting that loss of PHB negatively affects mitochondrial integrity. Inhibition of autophagy using Baf A or RNAi against ATG16L1 increased the percentage of control cells (Figure 5C, top panels) with mitochondrial membrane depolarization from ∼29% to ∼36% (Baf A) and ∼51% (siATG16L1). In cells with silenced PHB, treatment with Baf A increased the percentage of cells with mitochondrial membrane depolarization from ∼41% to ∼51% and siATG16L1 increased the percentage to ∼56% (Figure 5C, bottom panels). These results suggest that loss of PHB function induces mitochondrial dysfunction and that inhibition of autophagy increases the number of cells with altered mitochondrial function within the cell population.

### Silencing of PHB expression reduces cell viability

**Figure 6:**
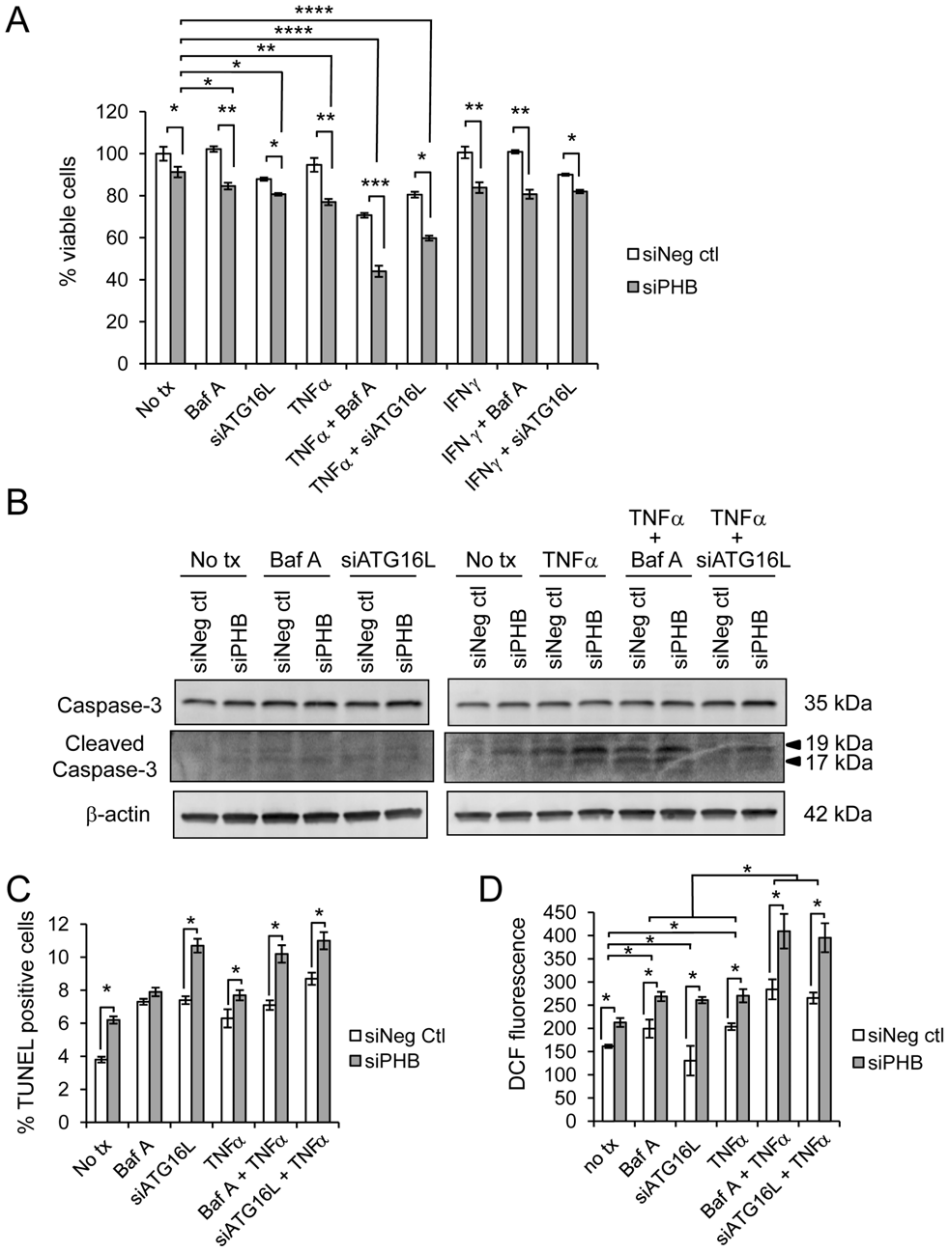
Silencing of PHB expression reduces cell viability. (A) Cytotoxicity as measured by LDH test of Caco2-BBE cells transfected with siRNA for 96 hours and treated as indicated for 18 hours. *P<0.05, **P<0.01, ***P<0.001; ****P<0.0001 vs. all other treatments; n = 3 experiments run in quadruplicate. (B) Representative Western blot showing cleaved Caspase-3 or β-actin (loading control) expression. (C) Percent TUNEL-positive Caco2-BBE cells. A minimum of 5 fields were counted for each treatment. *P<0.05. (D) DCF fluorescence was measured in Caco2-BBE cells transfected with siRNA for 96 hours and treated as indicated for 18 hours. Cells were loaded with DCFH-DA for 10 minutes and fluorescence was determined 10 minutes after loading. *P<0.05; n = 8.

Cell cytoxicity was assessed by measuring the release of lactate dehydrogenase. Across all treatments, Caco2-BBE cells transfected with siPHB were less viable than control cells (Figure 6A). Inhibition of autophagy using Baf A further decreased viability in siPHB-transfected cells compared to control cells. RNAi against the autophagy gene ATG16L1 reduced viability in both siPHB and siNeg ctl cells, but the effect was greater in siPHB cells. These results suggest that PHB knockdown reduces cell viability and that autophagy acts to promote cell survival. The effect of PHB knockdown to reduce cell viability was exacerbated by the pro-inflammatory cytokines TNFα and IFNγ (Figure 6A). IFNγ reduced viability in cells with PHB knockdown versus control cells, but in combination with autophagy inhibition IFNγ had no further effect than inhibition of autophagy alone. In contrast, TNFα dramatically reduced cell viability in siPHB-transfected cells with inhibited autophagy (Figure 6A), supporting the concept that the IFNγ autophagy pathway is distinct from that of TNFα. These results suggest that autophagy is important for cell survival when TNFα levels are high and when PHB expression is reduced, a scenario present during intestinal inflammation.

To determine whether cell death induced by PHB knockdown involves apoptosis, cleaved Caspase-3 protein expression and TUNEL staining were determined. Levels of cleaved Caspase-3 protein were increased during PHB knockdown and further increased by autophagy inhibition and TNFα treatment (Figure 6B). The percent TUNEL positive cells was increased during PHB knockdown and further increased by autophagy inhibition and TNFα treatment (Figure 6C), suggesting that cell death is at least partially due to apoptosis. To determine whether increased intracellular ROS is associated with increased cell death during autophagy inhibition and TNFα treatment, DCF fluorescence was measured in cells transfected with or without siPHB and treated with TNFα and autophagy inhibitors. Figure 6D shows that knockdown of PHB increased ROS levels for all treatment groups compared to control. TNFα treatment during inhibition of autophagy causes a further increase in intracellular ROS, which is further exaggerated in cells with PHB knockdown.

### Treatment with NAC, a ROS scavenger, prevents siPHB-induced mitochondrial stress-related autophagy

**Figure 7:**
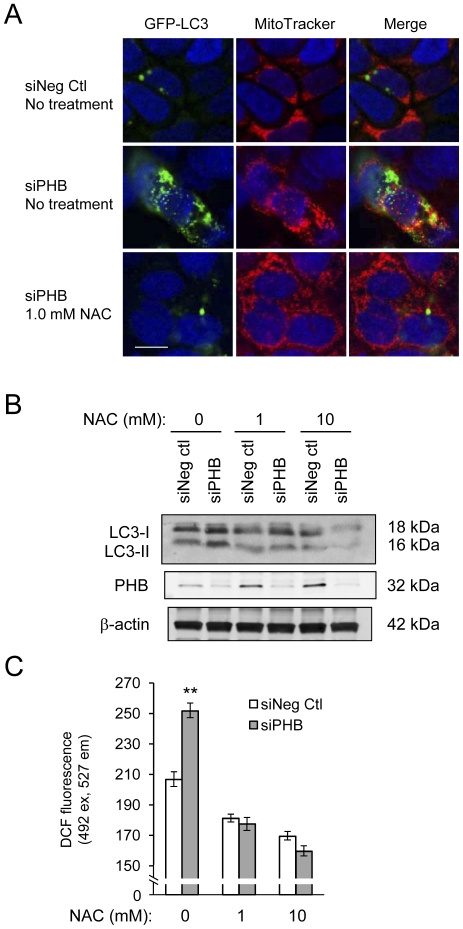
Treatment with NAC, a ROS scavenger, prevents siPHB-induced mitochondrial stress-related autophagy. (A) Caco2-BBE cells were co-transfected with siPHB or siNeg Ctl and GFP-LC3 (green fluorescence) and incubated with MitoTracker dye (red fluorescence, mitochondria). Cells were incubated with 1.0 mM NAC for 24 hours prior to collection. Merged confocal images demonstrate mitophagy (yellow fluorescence). Cells were stained with DAPI to visualize nuclei (blue). Bar = 10 µM. (B) Representative Western blot showing LC3I/LC3II or β-actin (loading control) expression. The data shown are a representative result from one of three experiments. (C) DCF fluorescence was measured in Caco2-BBE cells transfected with siPHB for 96 hours and treated with NAC for 18 hours. Cells were loaded with DCFH-DA for 10 minutes and fluorescence was determined 20 minutes after loading using a plate reader to assess intracellular ROS levels. **P<0.01, n = 8.

To determine whether mitochondria are indeed recycled during siPHB-induced autophagy, siPHB and GFP-LC3 co-transfected cells were labeled with MitoTracker, a mitochondria-specific dye (red fluorescence). Laser scanning confocal microscopy revealed significant co-localization of mitochondria with GFP autophagosomes in cells with silenced PHB compared to control cells (Figure 7A, upper panels versus middle panels), suggesting mitophagy. To determine whether increased intracellular ROS is the mechanism by which PHB knockdown induces autophagy, cells were treated with 1.0 mM NAC for 24 hours prior to collection. The addition of NAC prevented the increase in GFP-LC3 puncta, indicative of autophagosomes, by PHB knockdown and prevented the co-localization of GFP-LC3 with mitochondria (Figure 7A, lower panels).

Caco2-BBE cells were transfected with siPHB for 96 hours and treated with 1.0 mM or 10.0 mM NAC for 24 hours starting at 72 hours post-transfection. PHB knockdown stimulated the conversion of LC3I to LC3II compared to siNeg ctl cells (Figure 7B), indicating autophagy, as shown in Figure 3A. The addition of NAC prevented siPHB-induced conversion of LC3I to LC3II in a dose-dependent manner (Figure 7B). These results suggest that silencing of PHB expression increases intracellular oxidative stress, which subsequently induces mitophagy. DCF fluorescence was measured to ensure that NAC treatment reduced intracellar ROS (Figure 7C).

## Discussion

In addition to the emerging role of autophagy in controlling bacterial invasion [15], [53], [54], autophagy recycling represents the final tier of mitochondrial quality control. Multiple studies have reported mitochondrial dysfunction in Crohn's disease and ulcerative colitis [16], [17], [18], [19] and mouse models of colitis [20], [21]. Mitochondria are important regulators of autophagy and apoptosis. Exogenous ROS and cytokines such as TNFα, both of which are increased during IBD, promote cellular injury and autophagy via mitochondrial ROS generation [29], [30], [31]. We show here that PHB modulates autophagy in intestinal and colonic epithelial cells. Reduced PHB protein expression by RNA interference induces autophagy of mitochondria and treatment with NAC, a ROS scavenger, prevents siPHB-induced autophagy. These results suggest that loss of PHB expression induces autophagy via increased intracellular ROS. PHB knockdown induces mitochondrial membrane depolarization, suggesting mitochondrial damage, and increased intracellular ROS which is likely generated via dysfunctional respiration, all of which are exacerbated by inhibition of autophagy. Therefore, autophagy plays a protective role during conditions when PHB expression is low in intestinal epithelial cells.

We have previously shown that expression of PHB protein is reduced in mucosa during active inflammatory bowel disease and in Caco2-BBE cells treated with TNFα or exogenous oxidants [37], [42]. It has been demonstrated that TNFα and IFNγ induce autophagy in intestinal epithelium [31], [55] and that autophagy can attenuate inflammatory responses, thereby maintaining intestinal homeostasis [56]. PHB protein levels inversely correlated with TNFα and IFNγ-induced autophagy in Caco2-BBE and HCT116 cells. Furthermore, exogenous PHB expression reduced basal autophagy and TNFα-induced autophagy, suggesting that expression of PHB modulates the cellular autophagic response. One potential mechanism whereby PHB regulates *beclin-1*, the first mammalian gene shown to induce autophagy, is through the transcription factor E2F1. The *beclin-1* promoter contains a putative E2F1 binding site [57] and PHB has been shown to regulate E2F1 activity [35]. Future studies will investigate this possibility. It is compelling to speculate that reduced PHB expression coupled with dysfunctional autophagy, an emerging susceptibility trait in Crohn's disease [13], [14], [15], could render epithelial cells unable to recycle damaged mitochondria and thus they succumb to cell death. It has been shown that when less than 66% of the mitochondria within a cell are damaged, autophagy predominates to restore cell homeostasis, while apoptosis is triggered when the percentage increases to involve most of the mitochondrial population [58], [59]. The likely reason cell viability is decreased during PHB knockdown coupled with autophagy inhibition or TNFα treatment is that the threshold of damaged mitochondria exceeds the capacity of the cell.

Our finding that decreased PHB levels coupled with inhibited autophagy increased cell death has important clinical implications. Patients with inflammatory bowel disease exhibit decreased expression of PHB in intestinal epithelial cells [37], [41]. Dysfuntion in autophagy genes such as ATG16L1, IRGM, and LRRK2, are emerging as potential susceptibility traits in patients with inflammatory bowel disease [9], [10], [11], [12]. We speculate that decreased expression of PHB during active inflammatory bowel disease is a signal to the epithelial cell that there is inflammatory stress and that autophagy is subsequently induced to maintain cell viability and return homeostasis. Thus, these findings support an important role of autophagy in intestinal health and lend further insight into the mechanisms of dysfunctional autophagy via PHB in inflammatory bowel diseases. In the setting of PHB overexpression coupled with autophagy inhibition, ROS levels were not further increased and in fact are decreased during loss of ATG16L1. These data support the therapeutic concept of repletion of PHB levels in the setting of dysfunctional autophagy.

Basal autophagy was reduced by PHB overexpression in Caco2-BBE cells. We have shown previously that exogenous PHB expression in Caco2-BBE cells induced the expression of glutathione-S-transferase π, an antioxidant enzyme that catalyzes the conjugation of electrophiles to GSH [37], and modulates the activity of nuclear factor erythroid 2-related factor 2 (Nrf2), a transcriptional regulator of antioxidant response [42]. PHB overexpression may reduce basal autophagy through an increased antioxidant response on ROS production during normal physiological respiration. This issue warrants further investigation.

Although PHB protein levels inversely correlated with IFNγ-induced autophagy, overexpression of PHB in Caco2-BBE cells did not affect IFNγ-induced autophagy. This is in contrast to the response to TNFα. NF-κB, a major downstream signaling pathway of TNFα, has been implicated in modulating autophagy [60]. Our previous work demonstrated that exogenous PHB expression reduced basal and TNFα-stimulated NF-κB activation [42]. It is possible that this effect on NF-κB reduces TNFα-induced autophagy as compared to IFNγ since IFNγ is not a major activator of NF-κB. Furthermore, TNFα promotes autophagy via mitochondrial ROS generation [31]. TNFα treatment during inhibition of autophagy exacerbated intracellular ROS levels in cells with knockdown of PHB. Since PHB overexpression did not decrease IFNγ induced autophagy, this would suggest that the IFNγ autophagy pathway is distinct from that of TNFα. Exogenous PHB expression can likely prevent TNFα-induced autophagy since PHB overexpression reduces ROS generation. The mechanism whereby PHB overexpression dampens basal and TNFα-induced, but not IFNγ-induced, autophagy requires further investigation.

It is widely accepted that the tumor suppressor p53 regulates autophagy depending upon is subcellular localization [49]. Normal levels of p53 maintain a tonic inhibition of autophagy; autophagy is induced via a reduction in cytoplasmic p53 levels [61]. p53 can activate genes that induce autophagy including damage-regulated autophagy modulator (DRAM) and sestrins 1 and 2 [62]. Since Caco2-BBE cells have mutated p53, we included WT and p53 null HCT116 colonic epithelial cells to assess the involvement of p53 in autophagy mediated by PHB knockdown. PHB levels inversely correlated with TNFα- or IFNγ-induced autophagy regardless of p53 status. As previously described [52], p53^−/−^ HCT116 cells exhibited increased autophagy compared to WT cells. p53 knockout initially induces autophagy by ER stress followed by mitophagy [52]. Knockdown of PHB caused a further increase in autophagy in p53^−/−^ cells, suggesting that autophagy mediated by PHB knockdown is independent of p53. ER stress markers indicate that ER stress was not further increased upon PHB knockdown. We speculate that p53 null cells with PHB knockdown likely show more autophagy due to a combination of ER stress-induced autophagy (due to p53 knockout) and mitophagy (due to loss of PHB expression and p53 knockout).

In conclusion, we demonstrate that the mitochondrial protein PHB modulates autophagy in intestinal epithelial cells via intracellular ROS signaling. Decreased PHB expression coupled with inhibition of autophagy, renders intestinal epithelial cells unable to maintain cell homeostasis and susceptible to mitochondrial damage and cytotoxicity. These findings have elucidated a molecular pathway whereby increased ROS by decreased PHB may enhance inflammation in patients with inflammatory bowel disease.

## Materials and Methods

### Cell culture

The Caco2-BBE human intestinal epithelial cell line was used as in vitro model of polarized intestinal epithelium. The Caco2-BBE cell line was obtained from the American Type Culture Collection (ATCC; Manassas, VA). Since Caco2-BBE cells have mutated p53, wild-type (WT) and p53 null [51] HCT116 human colonic epithelial cells were utilized to assess the involvement of p53 in autophagy induction by PHB knockdown. Cells were grown in Dulbecco's modified Eagle's medium (DMEM; Caco2-BBE) or Modified McCoy's media (HCT116) supplemented with penicillin (40 mg/l), streptomycin (90 mg/l), and 10% fetal bovine serum. Caco2-BBE cells were plated on permeable supports to allow the cells to polarize (pore size, 0.4 µm; transwell-clear polyester membranes; Costas life science, Acton, MA), while HCT116 cells were plated on plastic. All experiments performed on Caco2-BBE cells were between passages 32 and 45.

### PHB knockdown and overexpression

Cells were transiently transfected with Stealth RNAi™ against PHB1 (5′-CAGAAUGUCAACAUCACACUGCGCA -3′) or Stealth RNAi™ siRNA Negative Control Med GC (Invitrogen, Carlsbad, CA) at 20 µm concentration. Caco2-BBEs were transfected using Amaxa® Cell Line Optimization Nucleofector® Kit T (Lonza, Basel, Switzerland), while HCT116 were transfected with LipofectAmine 2000 (Invitrogen). Cells were transfected with siRNA for 96 hours (Figure S1A–D). For PHB overexpression studies, Caco2-BBE cells were transiently transfected with either pEGFPN1 expression vector (V) or pEGFPN1-PHB (P) for 72 hours (generation of the pEGFPN1-PHB construct is described below).

### Autophagy activation and inhibition

Serum deprived cells were treated with 10 ng/ml recombinant human TNFα (R&D Systems, Minneapolis, MN) or 50 ng/ml recombinant human IFNγ (eBioscience, San Diego, CA) for 18 hours. When treating Caco2-BBE cells, TNFα was administered to the basolateral chamber, while IFNγ was administered to the apical and basolateral chambers. To inhibit autophagy, cells were treated with 100 nM Bafilomycin A_1_ (Baf A; Sigma-Aldrich, St. Louis, MO) 24 h prior to collection or co-transfected with 20 µm siATG16L1 (5′-AUUACUGCCAGAUAGGGAACCCUUG-3′). Efficiency of siRNA knockdown was assessed by Western blotting (Figure 5D). Cells were treated with 1.0 or 10.0 mM *N*-acetyl-*L*-cysteine (Sigma-Aldrich), a ROS scavenger, for 24 hours prior to collection.

### Generation of stably-transfected Caco2-BBE cell expressing pEGFPN1-PHB

A single PHB PCR product corresponding to the entire coding region of PHB (818 bp) was generated from Caco2-BBE cells using an antisense primer with a mutated PHB stop codon, underlined (5′-AATTGGATCCCCTCCCTGGGGCAGCTGGA-3′). The PCR product was ligated into pEGFPN1 vector (Clontech, Palo Alto, CA) using the Quick Ligation Kit (New England Biolabs, Ipswich, MA) and sequenced. Caco2-BBE cells were transfected with pEGFPN1-PHB or empty pEGFPN1 vector using Lipofectamine 2000 (Invitrogen) and the transfected clones were selected under 0.12% geneticin (Sigma-Aldrich), and fluorescent cells were isolated using flow cytometry (fluorescence-activated cell sorting).

### Western blot analysis

Total protein was collected from Caco2-BBE or HCT116 cells in lysis buffer containing 1% Triton X-100, 1% Nonidet P-40 (vol/vol), 1 mM EDTA, 1 mM sodium orthovanadate, 1 mM sodium fluoride, and 1 µl/ml mammalian protease inhibitor cocktail (BioExpress, Kaysville, UT) to obtain total protein extracts. The samples were separated by sodium dodecyl sulfate-polyacrylamide gel electrophoresis (SDS-PAGE) using laemmli's 2× SDS sample buffer and AnyKD™ gradient polyacrylamide gels (Bio-Rad, Hercules, CA) followed by electrotransfer to nitrocellulose membranes (Bio-Rad). Membranes were incubated with primary antibodies at 4°C overnight and subsequently incubated with corresponding peroxidase-conjugated secondary antibodies. Membranes were washed and immunoreactive proteins were detected using Amersham ECL Plus™ reagent (GE Healthcare, Piscataway, NJ). Blots were reprobed with anti-β-actin (Sigma-Aldrich) antibody. Mouse monoclonal PHB antibody was obtained from Thermo Fisher (Fremont, CA), mouse monoclonal GFP, mouse monoclonal ClpP, and rabbit polyclonal PKR antibodies from Santa Cruz Biotechnology (Santa Cruz, CA), rabbit polyclonal LC3 and ATG16L1 antibodies from Sigma-Aldrich, rabbit polyclonal Beclin-1, cleaved Caspase- 3, p53, Cpn60, and all ER stress antibodies from Cell Signaling Technology (Danvers, MA).

### Fluorescence microscopy

pSelect-NGFP-LC3 was purchased from InvivoGen (San Diego, CA). pSelect-GFP-LC3 was co-transfected into Caco2-BBE and HCT116 cells with either siNeg Ctl or siPHB for 96 hours. Cells were fixed in buffered 4% paraformaldehyde for 20 minutes, washed with phosphate buffered saline, and counterstained with DAPI to visualize nuclei. Stained cells were mounted in phenylenediamine/glycerol (1∶1) and analyzed by fluorescence microscopy (Zeiss Axioskope Plus).

### Confocal microscopy

Caco2-BBE cells grown to confluency on filters were transfected with pSelect-NGFP-LC3 for 96 hours and incubated with 100 nM MitoTracker dye (Invitrogen) for 15 minutes. A subset of cells was treated with 1.0 mM NAC for 24 hours prior to collection. Cells were fixed in buffered 4% paraformaldehyde for 20 minutes, washed with PBS, and counterstained with DAPI to visualize nuclei. Samples were mounted in *p*-phenylenediamine/glycerol (1∶1) and analyzed by confocal microscopy.

### DCF assay

As a measure of intracellular ROS, conversion of the nonionic, nonpolar 2′, 7′ –dichlorodihydrofluorescein diacetate (H_2_DCFDA: Invitrogen) to fluorescent 2′, 7′ –dichlorofluorescein (DCF) was measured. Caco2-BBE cells were loaded with 10 µM H_2_DCFDA for 10 minutes and fluorescence was quantitated 10, 20 and 30 minutes later according to the manufacturer's protocol using a plate reader.

### Cytotoxicity test

Lactate dehydrogenase (LDH) cytotoxicity detection kit (Clontech, Mountain View, CA) was used to measure cell viability. LDH determines the secretion of LDH into the culture medium from dead or membrane-damaged cells. Caco2-BBE cells transfected with siRNA for 96 hours and were treated with TNFα, IFNγ, or Baf A alone or in combination for 18 hours prior to collection. An aliquot of 100 µl of culture media was added to 100 µl of LDH reagent and % cytotoxicity and % viable cells were measured according to the manufacturer's protocol using a plate reader.

### Terminal deoxynucleotidyl transferase-mediated deoxyuridine triphosphate nick-end labeling (TUNEL) staining

TUNEL staining was performed on confluent Caco2-BBE cells as described by the manufacturer's protocol (Roche, Indianapolis, IN).

### FACS analysis of mitochondrial membrane potential

Mitoprobe™ JC-1 assay kit was used (Molecular Probes, Eugene, OR) to detect mitochondrial membrane depolarization. JC-1 is a cationic dye which gives orange emission upon aggregates in normal polarized mitochondria and in monomeric form gives green fluorescence in the depolarized mitochondria. Briefly, 4×10^5^ Caco2-BBE cells transfected with siNeg Ctl or siPHB for 96 hours were trypsinized, washed and resuspended in a final volume of 1 ml of warm media. Cells were stained with 2 µm JC-1 dye for 15 minutes at 37°C protected from light. After incubation, cells were washed and resuspended in 500 µl of phosphate buffered saline and were analyzed on Becton Dickinson FACS Canto II flow cytometer.

### Statistical analysis

Values are expressed as mean ± SEM. Comparisons between TNFα or IFNγ treatment or siPHB versus control were analyzed by unpaired Student's *t*-test. Comparisons between PHB RNAi and autophagy inhibition were analyzed by two-way analysis of variance to test for a significant interaction between PHB knockdown and impaired autophagy. Subsequent pair wise comparisons used Bonferroni post-hoc tests to test for significant differences between two particular groups. p<0.05 was considered statistically significant in all analyses.

## Supporting Information

Figure S1 **PHB knockdown does not induce a mitochondrial unfolded protein response in Caco2-BBE cells.** Representative Western blot showing expression of the mitochondrial stress proteins Cpn60, PKR, ClpP. PHB protein levels were assessed to determine efficiency of knockdown.
(TIF)

Figure S2 **ER stress markers are not further increased in p53-null HCT116 cells upon PHB knockdown.** Representative Western blots showing expression of various ER stess markers. Deletion of p53 causes an increase in ER stress as previously reported [52].
(TIF)
