# Supplementary figures and images for: Prohibitin 1 Modulates Mitochondrial Stress-Related Autophagy in Human Colonic Epithelial Cells
Source: PLoS One. 2012 Feb 17;7(2):e31231. doi: 10.1371/journal.pone.0031231 (PMC3281932; doi:10.1371/journal.pone.0031231)

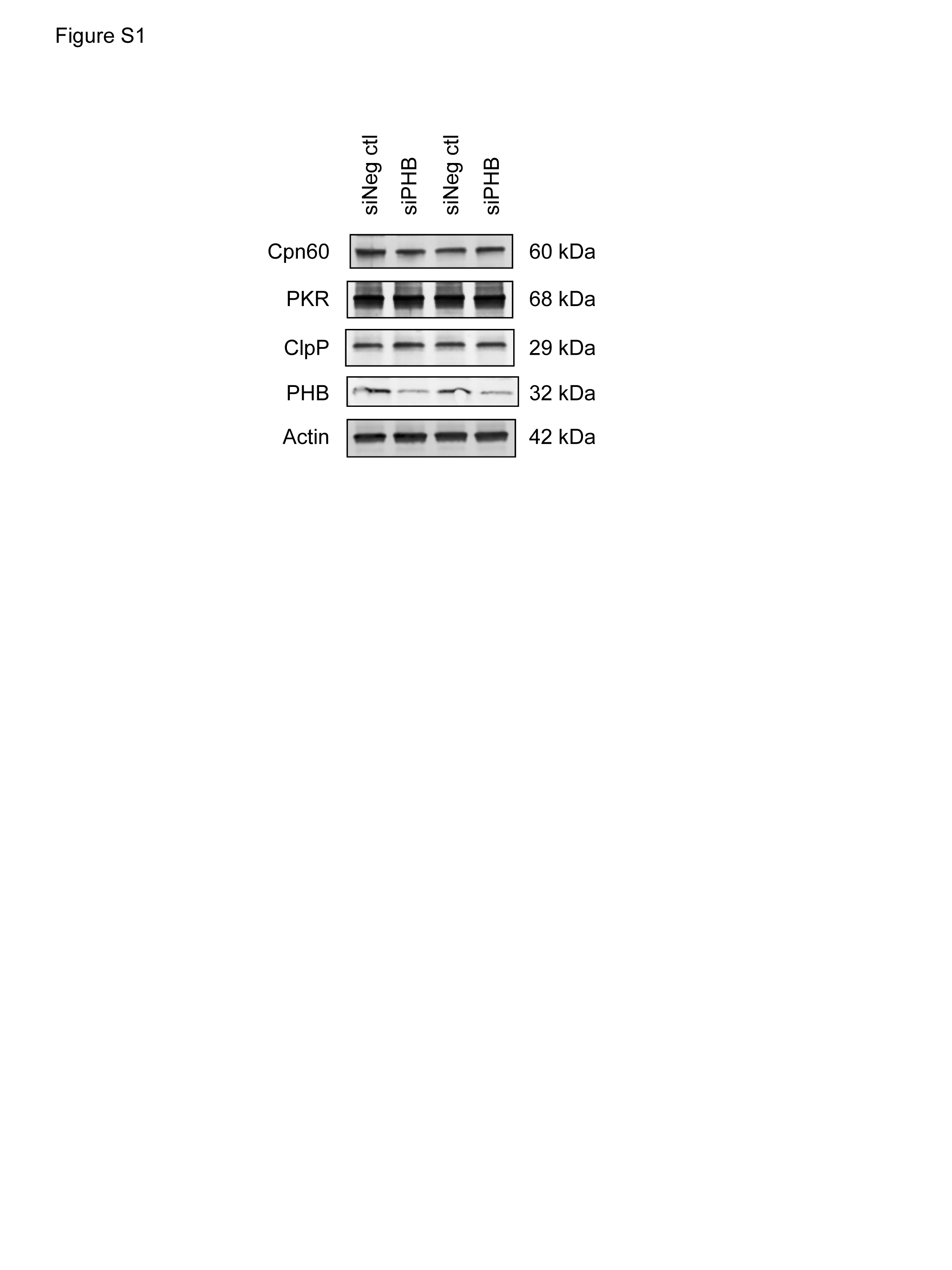

Supplement: Figure S1 — PHB knockdown does not induce a mitochondrial unfolded protein response in Caco2-BBE cells. Representative Western blot showing expression of the mitochondrial stress proteins Cpn60, PKR, ClpP. PHB protein levels were assessed to determine efficiency of knockdown. (TIF) [file pone.0031231.s001.tif]

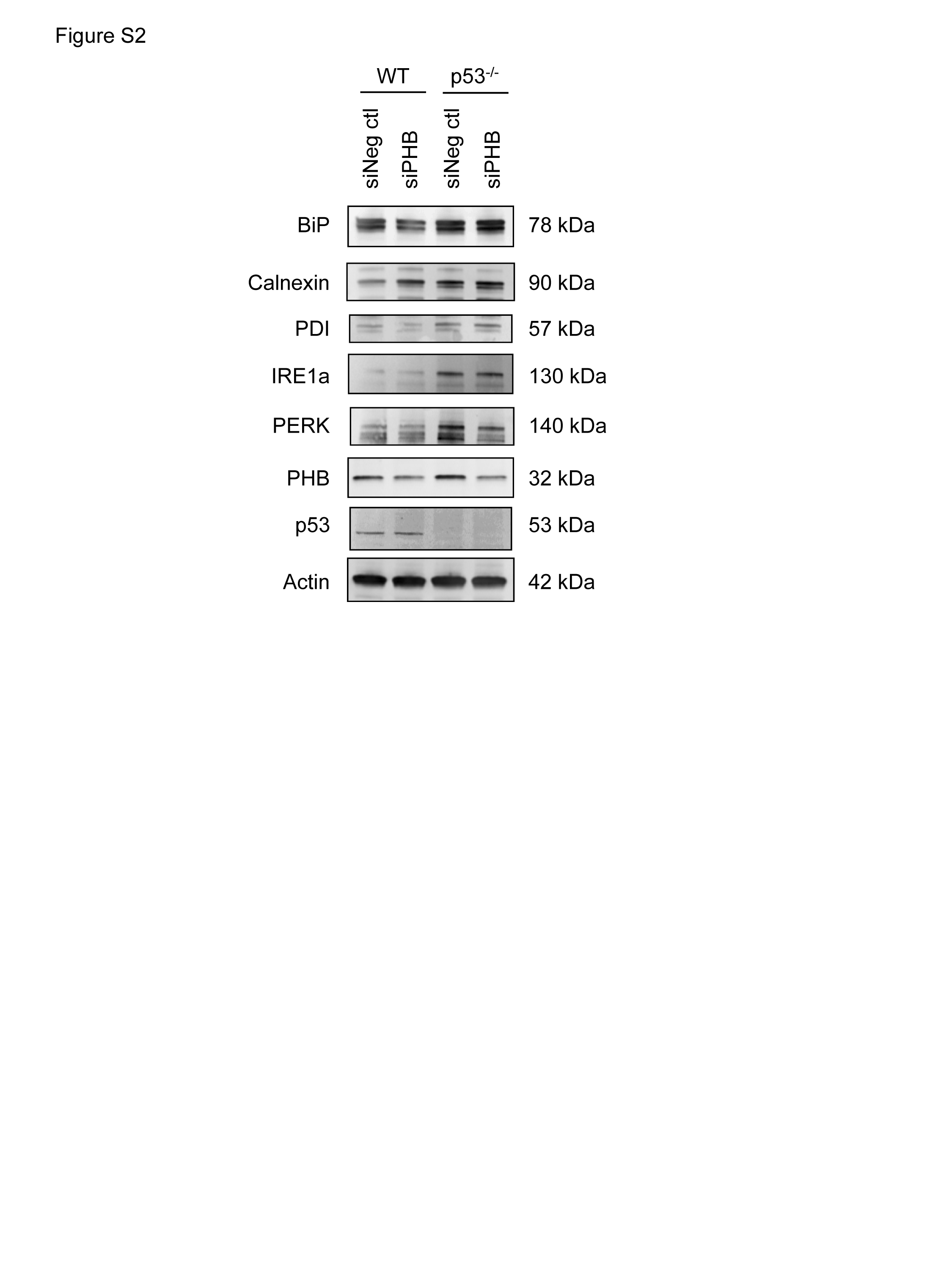

Supplement: Figure S2 — ER stress markers are not further increased in p53-null HCT116 cells upon PHB knockdown. Representative Western blots showing expression of various ER stess markers. Deletion of p53 causes an increase in ER stress as previously reported [52]. (TIF) [file pone.0031231.s002.tif]
